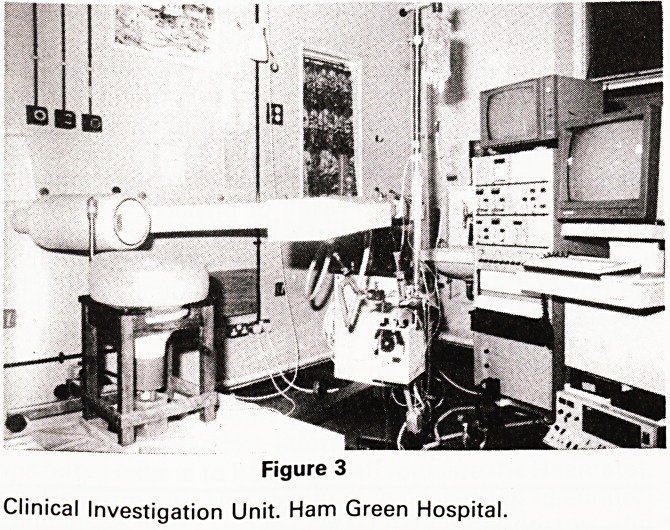# The Assessment of the Urological Opprobria

**Published:** 1986-12

**Authors:** R. C. L. Feneley

**Affiliations:** Consultant Urologist, Bristol Royal Infirmary and Southmead General Hospital


### The Assessment of the Urological Opprobria

**R. C. L. Feneley, M.Chir., FRCS**
Consultant Urologist, Bristol Royal Infirmary and Southmead General Hospital

'Opprobria medicinae' was an expression used by Dr. Long Fox in his Bradshaw Lecture of 1882, to describe 'those cases in which the exciting cause is not found, sometimes scarcely sought for'. In his lecture 'On the influence of the sympathetic on disease' he sought to explain common clinical manifestations by relating them to the condition of the sympathetic ganglion. 'Is not emotion,' he asked 'a brain discharge at least as much as epilepsy?' He cited the effect of emotion on the bladder as a cause of frequency of micturition, adding that this was one of the most usual results of terror and probably experienced by a large number of candidates for examination. According to Dr. Long Fox, this was caused by paralysis of the purely sympathetic nerves, thus postulating their inhibitory influence on bladder function. Disorders of micturition may aptly be described as the 'urological opprobria'; their clinical features are familiar yet their aetiology is invariably complex.

In his Presidential address to the B.M.A. in 1894, Dr. Long Fox drew attention to the enormous growth in opportunities for education in Bristol during the previous thirty years and it is of interest to examine his close association with the two leading figureheads, whose cooperation led to the foundation of the University College in 1876. When Long Fox went up to Balliol College, Oxford in 1850, his tutor was Benjamin Jowett, an intimidating character by all accounts, who was committed to the concept of developing a responsible elite. On qualifying in 1857 at the age of 25 years, Dr. Long Fox was appointed physician to the Bristol Royal Infirmary and in 1862 at the foundation of Clifton College he become physician to the school. This post brought him in close communication with the first headmaster, the Reverend John Percival, who would have been two years younger than Long Fox. In 1868 Percival formed the Association for Higher Education of Women and this led to the foundation of Clifton High School for Girls in 1877 and Redland High School in 1882. He later formed the Association for the Promotion of Evening Classes and in 1872 he published his notable proposal in a pamplet on 'The Connection of the Universities and the Great Towns'. He emphasised the need to raise 'the general life of the commercial classes to a higher level of cultivation and taste' and he suggested that each of the wealthier colleges at Oxford or Cambridge should convert two or more non-resident fellowships to be held in a great town, such as Birmingham, Bristol, Leeds or Liverpool. Such appointments should be held initially for a period of ten-to-twelve years, before being eligible for re-election. Benjamin Jowett, who was Master of Balliol at that time, gave his full support to Percival's concept and he persuaded New College, Oxford, to participate in the scheme. Both Percival and Jowett were committed to the principle that men and women should have equal status at the University College and this was a unique feature of the foundation. The University College of Bristol was opened in 1876 in converted buildings in Park Row, offering courses in fifteen subjects. Of the 99 pupils in the first term, there were 69 women and 30 men, but a much higher proportion of men attended the evening classes that were part of the academic curriculum.

Whilst the education scene was undergoing a profound metamorphosis, there were also fundamental changes in medical practice during Dr. Long Fox's lifetime. Surgical practice was to change dramatically with the introduction of anaesthesia in the 1840s. Until the nineteenth century, surgical principles had continued to be influenced by the teaching of Hippocrates and Galen. In the Oath, Hippocrates had stated 'I will not cut persons labouring under the stone, but will leave this to be done by practitioners of this work'. He taught that wounds of the bladder were invariably fatal, and hence any abdominal approach to the bladder was considered hazardous. For centuries, lithotomy had been the opprobrium of urological surgery. Lithotomists tended to be itinerant characters, probably owing to the high mortality of their trade. Jacques de Beaulieu, better known as Frère Jacques, had been apprenticed to a roving Italian surgeon Pauloni before he arrived in Paris in 1697. There he made his reputation by performing ten successful operations for stone at Fontainbleau, but then his fortunes changed and his next ten patients died, so he was forced to leave Paris to escape the repercussions. However, he returned after a short absence and he learned how to use the grooved sound with immense success. In 1703 he was summoned by the Maréchal de Lorges, who was suffering from a bladder stone. Before submitting himself to surgery, the Maréchal gathered 22 patients at his mansion and invited Frère Jacques to operate on them, which he did with complete success. The Maréchal then submitted himself to surgery, but subsequently died. By the time of his death in 1714, Frère Jacques had probably performed about 4,500 operations for stone in Europe.

Obstruction to the passage of urine was considered by Galen in the second century A.D. to be related to the fleshy excrescences in the urethra and this concept of carnosities and caruncles continued until John Hunter's studies in the latter part of the eighteenth century. In the sixteenth century Ambroise Paré, often called the father of modern surgery, published an account of the symptomotology of bladder disorders. He described the three impediments to the urine flow, namely dysuria – when there is pain in passing urine, strangury – when urine passes drop-by-drop and ischuria – when the urine is suppressed or stopped. His treatment of these conditions started with the application of fomentations and ointments to the perineum, but if these failed he recommended the passage of a leaden catheter with a rough button at its end, which could be thrust up and down to break and tear the caruncles.

Hyperplasia of the prostate as a cause of obstruction was recognised by John Hunter in 1788, but it was his brother-in-law Everard Home who published his work in 1811. By the time of Long Fox's undergraduate training, the clinician was advised to differentiate carefully between the empty and the full bladder, in order to distinguish between the diagnosis of congestion of the prostate and retention of urine respectively. Congestion of the prostate was treated by a regime of cupping and application of leeches to the perineum, hip baths twice daily, venesection, gruel enemas and tincture of hyoscyamus. Retention of urine required catheterisation twice daily until spontaneous voiding was re-established. If a stricture of the urethra prevented catheterisation, the complications could be sinister. Dr. Cock, surgeon to Guy's Hospital, wrote in 1852 that 'stricture may well be called the opprobrium of surgery' and he advocated transrectal catheterisation with a trocar and cannula. In that same year the Jacksonian prize was awarded to Sir Henry Thompson for his essay on 'Stricture of the urethra' and he received the same prize again in 1860, with an essay of 'Diseases of the prostate gland'.

A hundred years ago, in 1885, von Dittel performed the first planned partial prostatectomy. He operated on a physician who had found repeated catheterisations to be quite intolerable. A suprapubic tube had been inserted as an alternative method of treatment, but this failed to drain satisfactorily. To overcome the problem, von Dittel enlarged the suprapubic fistula and removed the middle lobe of the prostate with a snare. His patient died six days later from uremia. However, following this, a growing number of reports appeared on the subject of prostatic surgery.

Arthur Ferguson McGill (Fig. 1) performed the first partial prostatectomy in England at Leeds General Hospital in 1887. His patient was Edward Ward, aged 53, whose occupation was described as 'a smith'. He had originally consulted McGill five years previously, owing to pain on passing urine. However, McGill had sounded him but had found no evidence of stone. Later the patient developed incontinence of urine, for which he wore a bag, and at times he noted gravel to be present in the urine. For seven months, before admission to hospital, the patient had been catheterising himself every two hours, day and night. Following his admission, McGill sounded him again and detected stones on that occasion. On March 24th 1887, under ether anaesthesia, the bladder was opened and six very large stones were removed. According to the operation note, 'a large growth surrounding the internal orifice of the urethra, which was discovered soon after the introduction of the finger, was then tried to be removed. There was a good deal of haemorrhage from it. After several attempts by means of the ecraseur and forceps, the greater part was ultimately removed by means of Spencer Wells pedicle forceps, clamping the base and tearing it away.' McGill thought that he had removed a bladder tumour. Because the house surgeon was not available, his assistant was a surgical dresser, who also performed the histological examination. He reported that the tumour was in fact a prostatic adenoma. The student clearly relished the thought of being able to tell his chief that he had made a mistaken diagnosis, but when he told him the following day, McGill merely retorted 'why don't we always take the prostate out when it projects into the bladder?' In later years that student was to become Lord Moynihan.

**Figure 1**

Arthur Ferguson McGill.

The following year, in 1888, McGill performed the first complete enucleation of the prostate gland, with the three lobes surrounding the urethral passage. By 1890, Belfield of Chicago produced a critical review on 88 prostatic operations, which included perineal, suprapubic and combined perineal and suprapubic procedures. He stressed that the principle of the radical operation assumed:

1) that the cause of chronic retention is mechanical obstruction from an enlarged prostate
2) the obstacles are capable of removal
3) after such removal the bladder will resume its function of voluntary evacuation.

The introduction of prostatic surgery stimulated considerable controversy. When Freyer published his paper on the total extirpation of the prostate in 1901, Eugene Fuller of New York was indignant that Freyer had described the operation which he had published in 1895. It transpired that a colleague of Fuller, Ramon Guiteras, had passed through London in 1900 on his way to an international medical congress in Paris, where he was reading a paper on 'The present status of the treatment of prostatic hypertrophy in the United States'. Freyer met Guiteras, who explained Fuller's method of enucleating the prostate, and it was only following that meeting that Freyer performed his first operation. To assist the enucleation of the prostate, Freyer advised that the surgeon should grow the nail of the index finger long and then sharpen it, to which Fuller commented that this was Freyer's only contribution to the operation.

By the time of Dr. Long Fox's death in 1902, the era of prostatic surgery had been established and the technical performance of the operation presented the predominant challenge during the first half of the twentieth century. Thompson Walker reported the mortality in hospitals in England to vary between 12% and 23% during the period from 1918 to 1928. In his book on 'Retropubic Urinary Surgery', published in 1947, Millin referred to the triangular contest that had continued to exist up to that time between the protagonists of the three approaches to the prostate, namely the suprapubic, the perineal and the transurethral operations. He introduced the retropubic approach as a safe alternative method, which he considered would ease the convalescence and lead to more patients seeking earlier advice. His first patient in 1945 makes an interesting comparison with that of McGill's in 1887; he was a man of 72 years in gross uremia, who had been catheterising himself for three years, owing to complete urinary retention. Millin's prediction was correct. By 1967 about 24,000 prostatectomies were performed in England and Wales and the majority of these were either suprapubic or retropubic operations. Transurethral surgery was still developing, but the improvement in the telescopes, with the introduction of the Hopkins solid rod lens system in 1959, was to transform the situation. By 1982, around 34,000 prostatectomies were performed in England and Wales, and

**Figure 2**

Mosco and Pelicam's apparatus for recording bladder volume.

**Figure 3**

Clinical Investigation Unit. Ham Green Hospital.

of these 70% were performed by transurethral resection. In a report of 2,887 prostatectomies for benign enlargement in the Bristol area, Ball and Powell (1982) noted the mortality to be 1.6% for transurethral resection and 1.38% for open surgery.

During the past thirty years, the assessment of micturition disorders has been influenced by the introduction of urodynamic techniques. Objective measurement of the pressures within the bladder and urethral during filling, together with urine flow rates during voiding, have provided a different interpretation of the symptoms. Frequency and urgency of micturition, nocturia, hesitancy and a poor urinary stream had come to be recognised as 'prostatism', which is a misleading term both for the patient and the clinician. Assessment of the function of the lower urinary tract was not a new concept. In 1882, the year that Dr. Long Fox was delivering his Bradshaw Lecture, Mosso and Pellicani published their classic paper on the function of the bladder. With their simple apparatus (Fig. 2) they recorded the variations of bladder volume at constant pressure and they demonstrated the slow fluctuations of bladder volume, which occurred at times spontaneously and at others in attempts to micturate. These fluctuations occurred when the subject exerted emotional or intellectual effort and they concluded that the function of the bladder was closely related to the intensity of psyschological processes. With the advantage of modern sophisticated equipment (Fig. 3) the cause of the disorders of micturition continue to stimulate discussion and research. Does a functional disturbance of micturation eventually become an organic disease?

The opprobria, according to Dr. Long Fox, consisted of 'those cases in which the exciting cause is not found'. It is surely the search for the cause that stimulates advance in clinical practice. For centuries, surgery for stones, strictures and prostatic obstruction challenged and provoked the clinician's ingenuity. Clinical measurement has now introduced new techniques and new criteria for assessing micturition disorders and the results have emphasised the importance of physiological concepts in their management. Perhaps in the future the application of biofeedback will have a more major role in the management of many of these disorders.

### REFERENCES

CARLETON D. 'A university for Bristol: an informal history in text and pictures'. University of Bristol Press, 1984.

CLARK P. 'Moynihan, the urologist' Eur.Urol. 2: 48–53 (1976).

EVERARD HOME 'Practical Observations on treatment of disease of the prostate gland' 1811 published by London G. and Nicol M.

EDWARD COCK 'Remarks on the surgical operations usually adopted for retention of urine' 1852 Medico-Chirurgical Transactions **35** 153–187.

FOX E. L. 'The Bradshaw lecture on the influence of the Sympathetic on disease' (delivered before the Royal College of Physicians of London, August 18th 1882). British Medical Journal, 1882 **2** 343–348, 399–405.

MOSSO and PELLICANI 'Sur les fonctions de la vessie' 1882 Arch. Ital. Biol. 1 97–323.

WILLIAM T. BELFIELD 'Operations on the enlarged prostate with a tabulated summary of cases' 1890 The International Journal of the Medical Sciences Vol. 10 No. 5 439–452.

SIR PETER FREYER 'A clinical lecture on total extirpation of the prostate for radical cure of enlargement of the organ' 1901 Brit Med J. **2** 125–29.

EUGENE FULLER 'The question of priority in the adoption of the method of total enucleation suprapubically of the hypertrophied prostate' 1905 Ann. Surg. Vol 41 520–34.

TERENCE MILLIN 'Retropubic urinary surgery' 1947 published by E. and S. Livinstone, Edinburgh.

LEONARD J. T. MURPHY 'The history of urology' 1972 published by Charles C. Thomas (USA).

A. J. BALL and P. H. POWELL 'Prostatectomy trends in the Bristol area' 1982 British Journal of Urology **54** 539–541.

## Figures and Tables

**Figure 1 f1:**
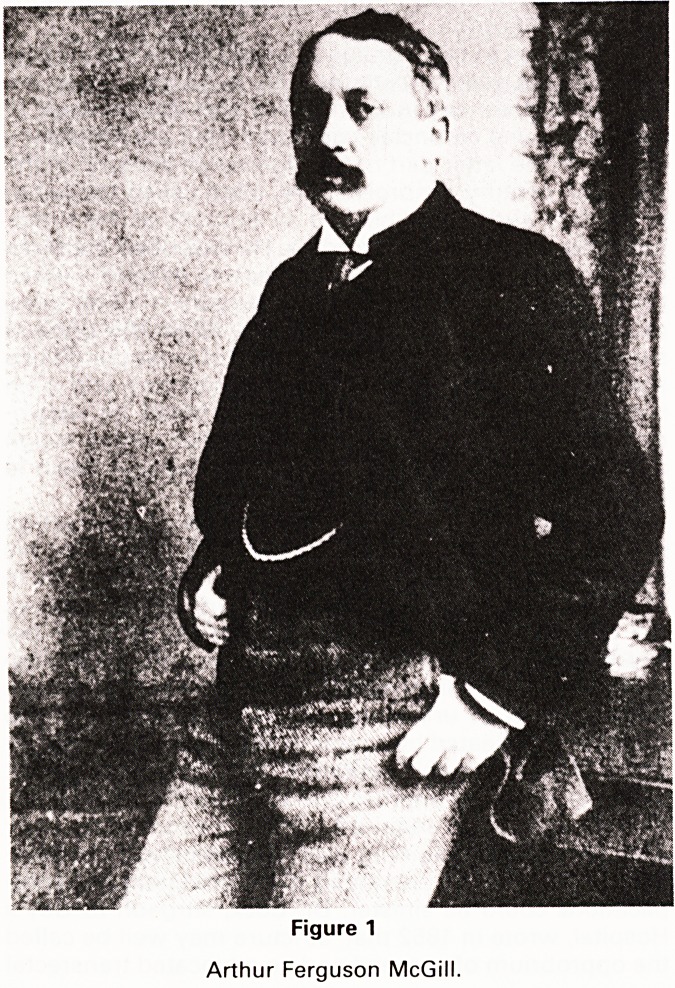


**Figure 2 f2:**
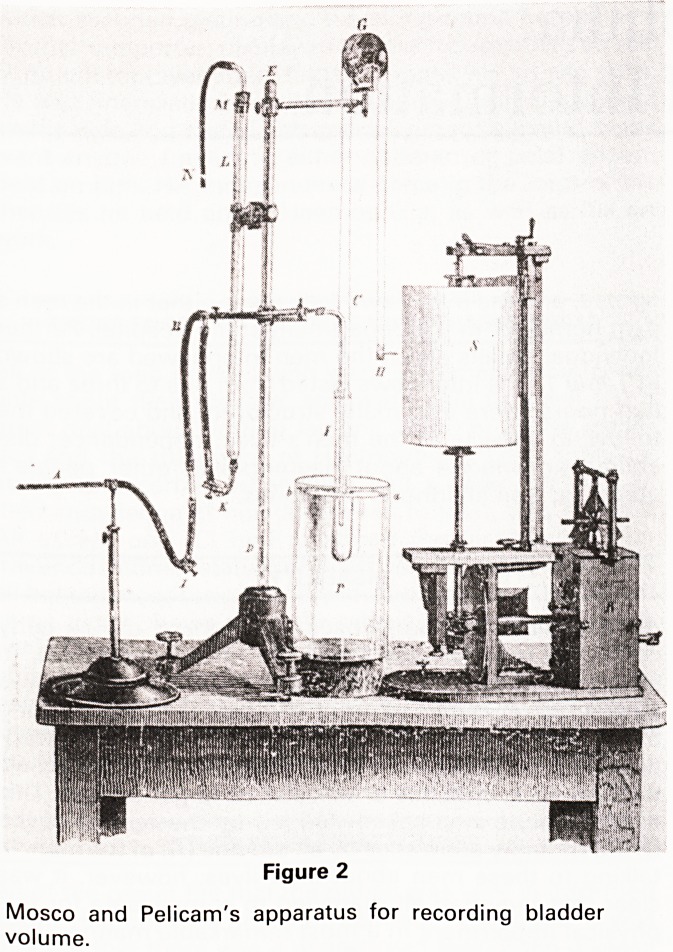


**Figure 3 f3:**